# Incidental Diagnosis of a Primary Pure “Mixed” Ovarian Carcinoid: Clinicopathological Report and Concise Review of the Recent Series

**DOI:** 10.1155/2024/5890300

**Published:** 2024-03-27

**Authors:** Demetrio Larraín, María José Gárate, Lidia Díaz, Iván Rojas

**Affiliations:** ^1^Department of Obstetrics and Gynecology, Clínica Santa María, Santiago, Chile; ^2^Department of Pathology, Clínica Santa María, Santiago, Chile

## Abstract

Primary ovarian carcinoid tumors (POCT) are well-differentiated neuroendocrine neoplasms and account for <0.1% of ovarian tumors. POCT usually arise in the context of mature cystic teratoma; however, pure primary ovarian carcinoids without teratomatous or mucinous elements are very rare. We present a case of a 54-year-old woman that underwent total laparoscopic hysterectomy and bilateral salpingo-oophorectomy because of endometrial hyperplasia without atypia. The ovaries were macroscopically normal. Pathology report revealed a primary ovarian carcinoid with mixed trabecular and insular growth patterns. Immunohistochemical was positive for chromogranine A, synaptophysin, and CDX2. The Ki-67 index was <1%. To exclude a metastatic carcinoid to the ovary, a Ga-68 PET/CT was performed. This case highlights the microscopic and immunohistochemical characteristics of pure POCT and potential pitfalls in their differentiation from metastatic carcinoids. In addition, differential characteristics of primary and metastatic ovarian carcinoids are discussed.

## 1. Introduction

Primary ovarian carcinoids are uncommon well-differentiated neuroendocrine neoplasms. They constitute less than 1% of all carcinoid tumors and 0.1% of all ovarian malignancies [1, 2]. Carcinoid tumors are derived from the cells of the diffuse neuroendocrine system and most often occur in the lungs and gastrointestinal tract [1]. Characteristically, these tumors have slow growth and usually display and indolent disease course [2]. However, the so-called carcinoid syndrome and the presence of metastatic disease have been described in patients with ovarian carcinoids [3–5].

Primary and secondary carcinoid tumors of the ovary have similar histological growth patterns but differ in their clinical course, treatment, and prognosis [3, 5]. While primary tumors tend to be unilateral, confined to the ovary, and mainly follow a benign course, metastatic carcinoids are usually bilateral and are associated with a poor prognosis [3, 5].

There are distinct subtypes of ovarian carcinoids based on their histopathologic pattern: insular, trabecular, strumal, and mucinous [4]. However, mixed forms (containing 2 or more types) have also been described [2, 6]. Most of primary ovarian carcinoids reported in the literature arose in association with mature cystic teratomas or mucinous cystadenomas [2, 3, 7, 8]. We present a case of an incidentally detected primary ovarian carcinoid tumor with trabecular and insular components without any teratomatous or mucinous components.

## 2. Case Report

**Figure 1:**
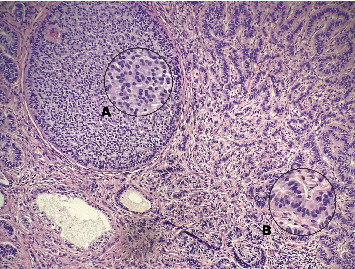
Histopathologic sections of the tumor with hematoxylin-eosin staining. The tumor exhibited mixed growth patterns: (A) insular and (B) trabecular (×200 magnification).

**Figure 2:**
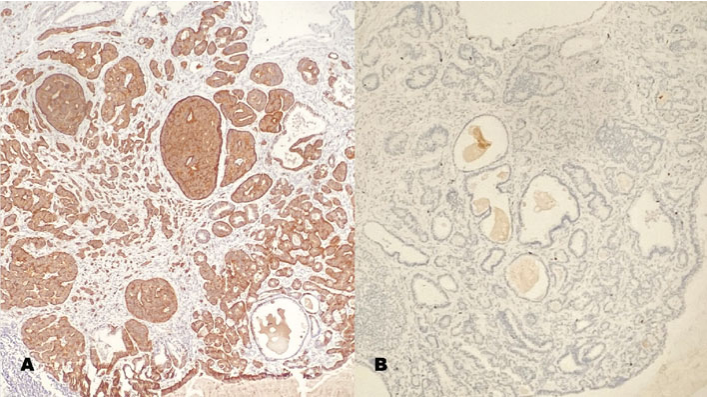
Immunohistochemical staining of tumor cells. (A) Diffuse positivity for synaptophysin. (B) The Ki-67 proliferation index < 1% was consistent with that of a carcinoid tumor (×200 magnification).

**Figure 3:**
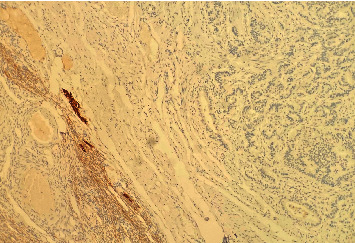
Negative immunostain for alpha-inhibin (×200 magnification).

A 54-year-old nulliparous woman was evaluated in our department for postmenopausal bleeding. Chronic stable medical problems included hypertension and hypothyroidism. There was no family history of breast, ovarian, uterine, or colon cancer. Transvaginal ultrasonography revealed two small intramural myomas and focal adenomyosis. Endometrial thickness was 4 mm, and both adnexa were normal. She underwent endometrial Pipelle sampling that demonstrated an endometrial hyperplasia without atypia. After counseling on potential treatment options, the patient opted for the hysterectomy. A total laparoscopic hysterectomy and bilateral salpingo-oophorectomy were performed without complications. The patient stayed in the hospital for one day and was discharge home without any complications. The pathology report confirmed the endometrial hyperplasia without atypia and revealed the presence of a 0.5 × 0.4 cm intraparenchymatous carcinoid tumor in the right ovary. Macroscopically, the ovaries seemed normal, and the ovarian capsules were intact. Microscopic examination of the affected ovary showed tumor cell ribbons and nests, surrounded by thick fibrous stroma, exhibiting mixed growth patterns, particularly of the trabecular and focal insular types (Figure 1). Lymphovascular space invasion and ovarian surface involvement were not identified. Contralateral ovary was normal. No teromatous or thyroid tissues were identified in any ovary. Immunohistochemical study showed positive staining for chromogranin A and synaptophysin (Figure 2(A)), confirming the neuroendocrine origin of the tumor. Other immunohistochemical markers included positive CDX2, Ki67 proliferative index < 1%, and negative alpha-inhibin (Figures 2(B) and 3). After establishing the pathological diagnosis, the patient underwent a gallium-68 DOTATATE PET/CT to evaluate for distant metastases or a different potential primary site. There was no evidence neither distant metastases nor another suspected primary site. Four months postoperatively, the patient is doing well and completely asymptomatic.

## 3. Discussion

The clinical characteristics and management of patients with primary ovarian carcinoids mainly originate from case reports and small retrospective cohort studies (Table 1). No recommended treatment guidelines are currently available for primary ovarian neuroendocrine carcinoid tumors. Although there is no consensus about the predominant histological type among primary ovarian carcinoids, available data shows that the trabecular form is rare and often associated with mature cystic teratomas or mucinous cystadenomas [2, 8, 9]. Potential differences in biological behavior among the four subtypes have been reported in the literature. Strumal carcinoids rarely present metastatic disease [10], but nonstrumal subtypes seem to have worse prognosis [11]. Davis et al. [11] reported three deaths due to nonstrumal primary ovarian carcinoids in 13 patients, including one trabecular carcinoid in FIGO stage I disease.

Ovarian carcinoids are known to cause carcinoid syndrome in less than one-third of patients, independent of the presence of metastases (Table 1). Theoretically, serotonin-like substances are released directly into the systemic circulation through the ovarian venous system, bypassing hepatic inactivation. In contrast, gastrointestinal carcinoids lack direct access to the vena cava and are reported to cause carcinoid syndrome infrequently, unless there is metastatic liver involvement [11]. While carcinoid symptoms are relatively frequent in insular carcinoids, primary trabecular ovarian carcinoids have rarely been associated with the presence of the carcinoid syndrome [7].

Similar to our case, most patients with ovarian carcinoid are asymptomatic. Usually, primary ovarian carcinoids are found incidentally on cross-sectional or ultrasound imaging while working up other gynecological symptoms. In other cases, carcinoids are diagnosed as an incidental histological finding while undergoing gynecological surgery for benign pathology [2, 3, 5].

Accurate diagnosis and classification of carcinoid ovarian tumors is challenging and requires careful evaluation by an experienced pathologist since they usually represent small foci concealed within teratoma or cystadenoma components. In a recent study, mean size of carcinoid component was only 0.4 cm [5], just like in our case. Therefore, it can be easily overlooked. Moreover, immunohistochemistry is necessary to the correct identification of carcinoids since they typically express markers of neuroendocrine differentiation such as synaptophysin and chromogranin A [12]. The negative immunostain for alpha-inhibin excluded the possibility of sex cord tumor.

Comparing to primary ovarian carcinoids, neuroendocrine tumors from extraovarian origin with metastasis to the ovary are more common, and they are mostly insular type. Therefore, the diagnosis of ovarian carcinoid needs careful evaluation of the contralateral ovary and gastrointestinal tract to exclude the presence of a carcinoid tumor metastatic to the ovary, especially when teratomatous or mucinous components are absent [2, 12]. These distinction is crucial since it has both clinical and prognostic implications.

The value of tumor morphology should not be overlooked, as unilaterality, small size, the lack of multinodular growth, and the presence of associated teratomatous elements strongly favor a primary ovarian origin. On the other hand, features in favor of metastatic carcinoids include the absence of teratomatous elements, bilateral ovarian involvement, nodular growth pattern, and prominent lymphovascular space invasion and the presence of extraovarian metastases [2, 4]. Therefore, problems arise when faced a unilateral ovarian carcinoid tumor without other teratomatous elements and any of the features which are suggestive of a metastasis, such as it occurred in our case.

Immunohistochemical expression of CDX2 has been studied for such distinction. Desouki et al. [13] evaluated CDX2 expression in differentiating primary ovarian carcinoids from metastatic carcinoids to the ovary from primary gastrointestinal origin. The authors found that primary ovarian carcinoids without teratomatous elements did not express CDX2, whereas metastatic carcinoids expressed the marker. Furthermore, although 19% of teratoma-associated teratomas expressed CDX2, the authors argued that the presence of teratomatous elements strongly favors a primary ovarian origin [13]. Therefore, they concluded that CDX2 expression can be a useful marker distinguishing primary ovarian carcinoids from metastatic gastrointestinal carcinoids [13]. Based on that findings, the expression of CDX2 in our case could favor a secondary ovarian carcinoid. However, another study demonstrated diffuse CDX2 staining among insular and mucinous carcinoids of primary ovarian origin, as well as by those of gastrointestinal origin [14]. Conversely, CDX2 was not expressed by trabecular carcinoids of either primary ovarian or intestinal origin [14]. Considering that other possible primary carcinoid sites, such as the lungs and rectum, do not express CDX2, the CDX2 staining profile is not helpful and could be a potential pitfall in reliably distinguishing a primary ovarian carcinoid from a metastatic one [14]. In the present case, an insular carcinoid component was identified; therefore, CDX2 positivity was not surprising.

In a recent study, the Ki-67 proliferative index was evaluated in both primary and secondary ovarian carcinoids and correlated with clinical outcomes [15]. The authors proposed that Ki-67 index can be used to distinguish primary from metastatic ovarian carcinoids since secondary tumors usually exhibit a high Ki-67 index compared to primary ones (median 2.3 and 9.7, respectively) [15]. In fact, Ki-67 index in primary ovarian carcinoid tumors of insular, trabecular, and strumal types is usually less than 1% [4]. Furthermore, Ki-67 index can be used as a prognostic parameter as it is significantly associated with patient survival in both primary and metastatic ovarian carcinoids [15].

Therefore, since there are no histomorphologic criteria or immunophenotype which reliably distinguish between a primary and secondary insular, trabecular, or mucinous carcinoid tumors within the ovary, there will be cases in which such distinction will be impossible based solely on histopathologic and immunohistochemical analysis. In such cases, clinicopathological correlation and radiological investigations are needed. Specialist radionuclide imaging, such as galium-68 DOTATATE PET-CT, is usually useful in evaluating extraovarian primary neuroendocrine tumors, and they also give valuable staging information [2].

Based on the aforementioned data, we concluded that we faced a primary ovarian carcinoid, and no further follow-up was performed.

## Figures and Tables

**Table 1:** Clinicopathological data from recent primary ovarian carcinoid series.

Author (year) (ref)	*n*	Median age	Carcinoid syndrome	FIGO stage	Most frequent subtype	Teratoma-associated	Design
McGrath (2016) [3]	18	48.5 y	11%	I (78%) III (11%) IV (11%)	Insular	44%	Retrospective
Kong (2023) [5]	56	42 y	0%	I (98.2) III (1.8)	Strumal	NA	Retrospective
Yan (2021) [8]	4	52 y	0%	I (100%)	Strumal	100%	Retrospective
Davis (1996) [11]	17	55 y	29%	I (65%) III (17.5%) IV (17.5%)	Insular	53%	Retrospective
Desouki (2013) [13]	46	52 y	NA	NA	Insular	35%	Retrospective
Rabban (2009) [14]	16	52 y	NA	NA	Insular/strumal	63%	Retrospective
Zhang (2018) [15]	9	68 y	33%	NA	Insular/strumal	78%	Retrospective
